# Attenuation of Hemodynamic Responses to Laryngoscopy and Tracheal Intubation: Propacetamol versus Lidocaine—A Randomized Clinical Trial

**DOI:** 10.1155/2014/170247

**Published:** 2014-04-13

**Authors:** Ali Kord Valeshabad, Omid Nabavian, Keramat Nourijelyani, Hadi Kord, Hossein Vafainejad, Reza Kord Valeshabad, Ali Reza Feili, Mehdi Rezaei, Hamed Darabi, Mohammad Koohkan, Poorya Golbinimofrad, Samira Jafari

**Affiliations:** ^1^Department of Ophthalmology and Visual Sciences, University of Illinois at Chicago, IL 60612, USA; ^2^Department of Anaesthesiology, Tehran University of Medical Sciences, P.O. Box 14155-6447, Tehran, Iran; ^3^Department of Epidemiology of Biostatistics, Tehran University of Medical Sciences, P.O. Box 14155-6447, Tehran, Iran; ^4^Department of Dermatology, Golestan University of Medical Sciences, Gorgan 4934174515, Iran; ^5^Zainaldin Martyr Research Center, Gorgan University of Agricultural Sciences and Natural Resources, P.O. Box 15739-49138, Gorgan, Iran; ^6^Center for Cardiovascular Research, University of Illinois at Chicago, 835 S. Wolcott Avenue, Chicago, IL 60612, USA

## Abstract

The purpose of this study is to assess the effects of propacetamol on attenuating hemodynamic responses subsequent laryngoscopy and tracheal intubation compared to lidocaine. In this randomized clinical trial, 62 patients with the American Anesthesiologists Society (ASA) class I/II who required laryngoscopy and tracheal intubation for elective surgery were assigned to receive propacetamol 2 g/I.V./infusion (group P) or lidocaine 1.5 mg/kg (group L) prior to laryngoscopy. Systolic and diastolic blood pressures (SBP, DBP), mean arterial pressure (MAP), and heart rate (HR) were recorded at baseline, before laryngoscopy and within nine minutes after intubation. In both groups P and L, MAP increased after laryngoscopy and the changes were statistically significant (*P* < 0.001). There were significant changes of HR in both groups after intubation (*P* < 0.02), but the trend of changes was different between two groups (*P* < 0.001). In group L, HR increased after intubation and its change was statistically significant within 9 minutes after intubation (*P* < 0.001), while in group P, HR remained stable after intubation (*P* = 0.8). Propacetamol 2 gr one hour prior intubation attenuates heart rate responses after laryngoscopy but is not effective to prevent acute alterations in blood pressure after intubation.

## 1. Introduction

Propacetamol [4-(acetamido)phenyl N,N-diethylglycinate] is a prodrug, which is quickly hydrolyzed by plasma esterase to vigorous paracetamol; 1 gr propacetamol metabolized to 500 mg paracetamol [1]. It has an onset of about half an hour to an hour, has a half-life of one to four hours, and has duration effect of six to eight hours. Its optimal effects appear about one hour after injection and its maximum recommended dose in adults is 4 grams per day. This drug inhibits prostaglandins synthesis in the central nervous system and also blocks pain impulses peripherally and has antipyretic effects through hypothalamus [2]. This drug is safe, cost effective and its beneficial effects for pain management and reducing opioids amounts have been confirmed in patients who underwent dental, orthopaedic and gynaecologic surgeries [3–8].

Laryngoscopy and tracheal intubation are among the most painful processes carried out on the human body which are associated with acute hemodynamic responses, lasting for at least ten minutes [3–9]. Sympathoadrenal stimulation and subsequent catecholamine release may partially contribute to this hemodynamic instability, which is typically signified by an increase in heart rate (HR) and blood pressure (BP) [10]; however, the main mechanism is not clearly defined. These acute changes in hemodynamic status are particularly significant in patients with preexisting predisposing situations like hypertension, myocardial infarction, myocardial malfunction, and cardiovascular diseases [11, 12].

Various techniques have been examined for attenuating hemodynamic responses to laryngoscopy and tracheal intubation, including deeper anaesthesia [10], and numerous drugs, such as beta blockers [13, 14], calcium channel blockers [15], opioids [16–18], sodium channel blockers [18, 19], vasodilators [20], and alpha agonists [21]. Opioids are the most commonly used drugs with satisfactory outcomes for preventing hemodynamic subsequence of intubation. These drugs are not cost effective, however, and are associated with some unfavourable complications such as nausea, vomiting, consumedly sedation, and respiratory depression [22]. Therefore, there has been a growing trend to find an effective substitute to reduce these side effects as much as possible [23, 24]. However, the beneficial effects of propacetamol have been confirmed for postoperative pain management. The purpose of this study is to assess the effects of propacetamol on attenuating hemodynamic responses subsequent laryngoscopy and tracheal intubation compared to lidocaine, which is routinely used in most of operation rooms.

## 2. Subjects and Methods

### 2.1. Study Design, Inclusion and Exclusion Criteria

This single-blind randomized clinical trial was conducted between January 2010 and September 2011. The study protocol was approved by the ethical committee of Tehran University of Medical Sciences (TUMS). The cases were selected among the patients who underwent elective gynecologic and reconstructive surgeries in two operating rooms of Imam Khomeini Hospital, in Tehran, Iran. These cases had laryngoscopy and tracheal intubation before the surgery. The inclusion criteria were patients between the ages of 20 and 60 as well as American Anesthesiologists society (ASA) classes I and II. Patients with preexisting conditions were not included, namely, hard intubation (previous history of hard intubation, laryngoscopic view >II, body mass index >30, micrognathia, and macroglossia), diabetic patients with autonomic dysfunction, patients with renal and liver dysfunction, patients with a history of cardiovascular disease, hypertension, cerebrovascular diseases, respiratory disease (e.g., asthma), pheochromocytoma, cushing's syndrome, opium addiction, propacetamol or lidocaine contraindications, and hypersensitivity, and patients who needed emergent surgery.

### 2.2. Randomization and Study Protocol

The study protocol was explained in detail for those who met the inclusion criteria. Those who agreed and filled informed consent were enrolled in the study. A specific code was considered for each patient, and they were randomly allocated to two study groups, propacetamol (P) and lidocaine (L).

In the operating room setting, a peripheral I.V. line was drawn from the patient's hand, and a ringer lactate (2 cc/kg) was administered. A standard monitoring system, including electrocardiogram and noninvasive blood pressure and pulse oximetry, was set for each participant. One hour preceding anaesthesia induction, patients in group P received propacetamol 2 g/IV/infusion diluted in 100 cc of normal saline (N.S). Patients in group L received lidocaine (1.5 mg/kg) two minutes before anesthesia. The grouping was concealed from anesthesiologist performed intubation and also from patients who participated in the study. Only the researcher who recorded hemodynamic variables at studied time points was aware of the grouping that was different from the person who analyzed the data.

The routine protocol for anaesthesia induction was similarly applied for both groups using I.V. administration of midazolam 0.02 mg/kg, fentanyl 2 *μ*g/kg, sodium thiopental 5 mg/kg, and atracurium 0.5 mg/kg. Three minutes afterwards, laryngoscopy and tracheal intubation were performed by two anesthesiologists, using tracheal tubes number 7–7.5 for men and number 6.5–7 for women. Anesthesia was maintained based on the operation by means of isoflurane with or without N_2_O and fentanyl 50 *μ*g each 30 minutes.

Hemodynamic status, including systolic and diastolic blood pressures (SBP, DBP), mean arterial pressure (MAP), and heart rate were recorded in a prepared check list for each individual in six time points, before drug administration (as baseline), after drug administration (before laryngoscopy), and at 1, 3, 6, and 9 minutes after tracheal intubation.

### 2.3. Primary and Secondary Outcomes

The primary outcome was to determine the effects of groups (L versus P) and time (before intubation and at 1, 3, 6, and 9 minutes after intubation) on MAP and HR. Secondary outcomes were assessment of changes in SBP, DBP, MAP, and HR before and after drug administration, and also their changes within the 9 minutes after laryngoscopy. The incidence rates of tachycardia (HR > 120 beat/min) and bradycardia (HR < 60 beat/min), hypertension (MAP > 120 mmHg), and hypotension (MAP < 70 mmHg) were assessed and compared between two groups.

### 2.4. Statistical Analysis

Results were reported as mean ± standard deviation (SD) for quantitative variables and percentages for categorical variables. SBP, DBP, MAP, and HR were compared using the unpaired *t*-test between two studied groups. The changes of these variables after drug administration were compared assessed using a paired test. A two-way analysis of variance (ANOVA) with repeated measures was conducted to determine the effects of groups (L versus P) and time (before intubation and at 1, 3, 6, and 9 minutes after intubation) on MAP and HR. In addition, a repeated measures one-way ANOVA was performed in each group to determine the effects of time on MAP and HR. Statistical analysis was performed using SPSS version 21 (SPSS Inc, Chicago, IL, USA) and statistical significance was accepted at *P* ≤ 0.05.

## 3. Results

**Figure 1:**
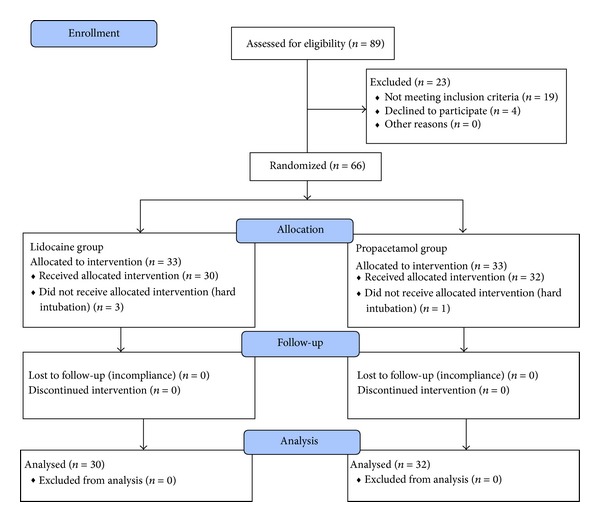
Study diagram.

Of 89 initial patients, 66 patients (Male 32) with the mean age of 41 ± 12 years (range: 20–60 years), who met inclusion criteria, were selected for study procedure and equally divided into two groups L and P. In total, four patients were removed from the study due to hard intubation (3 patients in group L and 1 patient in placebo group).  Figure 1 shows study fellow diagram.

Patients' demographic data in each group is shown in Table 1. Two groups did not have significant difference for age, sex, weight, height, ASA class, and endoscopic view (*P* ≥ 0.2). Hemodynamic status including SBP, DBP, MAP, and HR before and after drug administration (before laryngoscopy) has been summarized in Table 2. SBP, DBP, MAP, and HR were similar between two groups before drug administration (*P* ≥ 0.2). In both groups SBP, DBP, and MAP decreased after drug administration (*P* < 0.001). In group P HR decreased after drug administration (*P* < 0.001), while in group L it did not change significantly (*P* = 0.2).

**Figure 2:**
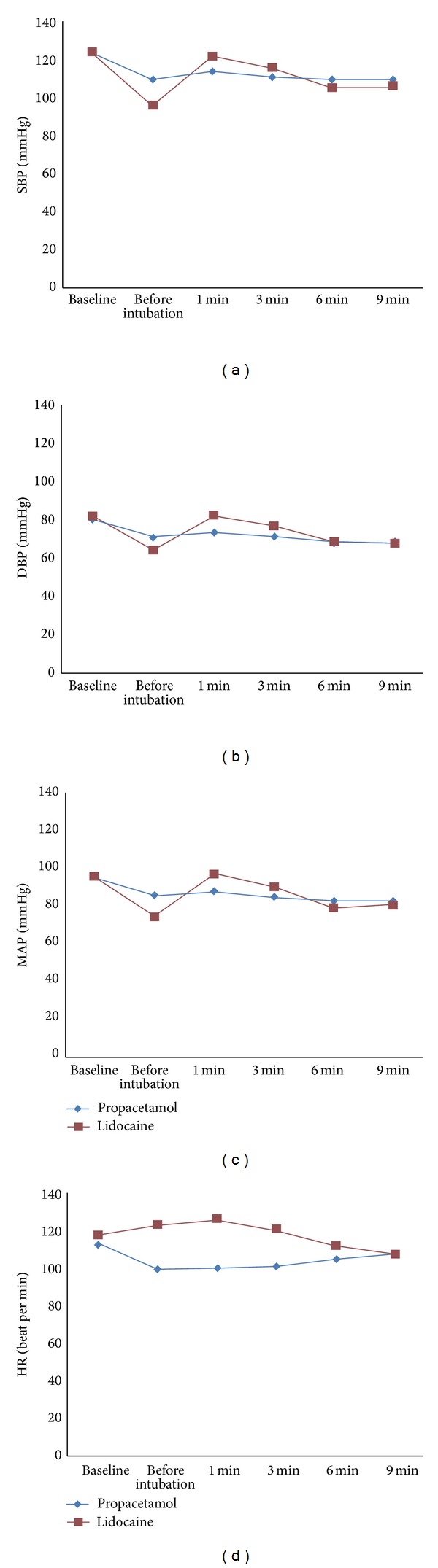
Hemodynamic status including systolic, diastolic, and mean arterial blood pressures (SBP, DBP, MAP) and heart rate (HR) within 9 minutes after tracheal intubation in each studied group.

The trend of SBP, DBP, MAP, and HR changes in each group has been shown in Figure 2. There was a significant effect of time on MAP (*P* < 0.001), but there was not a significant difference between two groups over the time points after intubation (*P* = 0.8). In both groups P and L, MAP increased after laryngoscopy and the changes were statistically significant (*P* < 0.001). There were significant effects of groups (P versus L) and time on HR after intubation (*P* ≤ 0.02). There were significant changes of HR in both groups after intubation, but the trend of changes was not the same between two groups. In group L, HR increased after intubation and its change was statistically significant within 9 minutes after intubation (*P* < 0.001). In group P, HR remained stable within the 9 minutes after intubation (*P* = 0.8).

The incidence rates of hypertension (3.0% versus 20.0%, *P* = 0.034), hypotension (0.0% versus 20%, *P* = 0.007), and tachycardia (3.0% versus 20.0%, *P* = 0.034) were significantly lower in group P as compared to group L (*P* < 0.05).

## 4. Discussion

In the present study, our findings showed beneficial effects of preoperative administration of propacetamol on attenuation of heart rate responses after tracheal intubation compared to lidocaine. None of the lidocaine or propacetamol was effective to attenuate blood pressure responses after laryngoscopy and changes of MAP after laryngoscopy were significant in both groups.

Although favorable effects of propacetamol on postoperative pain management have been well documented [3–8], there is no published evidence about its preventive effects on hemodynamic stimulations after tracheal intubation. However, there are some reports about the effects of acetaminophen on hemodynamic changes in subjects with a regular consumption of acetaminophen. These alterations include increase in blood pressure and cardiovascular events in subjects consuming acetaminophen [25–28]. In one two-week randomized crossover trial, acetaminophen (1 g three times daily) and not placebo increased systolic and diastolic blood pressure as determined by ambulatory monitoring [28]. However, other authors had reverse opinions [29, 30]. It seems that a single injection of propacetamol could not develop these side effects; however, it was not to attenuate the responses after laryngoscopy as well.

The exact underlying mechanism of the effect of propacetamol on cardiovascular responses is not clear. It might be attributed to its analgesic action mediated by the antiprostaglandin effect. Similar to NSAID and aspirin, propacetamol inhibits cyclooxygenase, prostaglandin H2 synthase in its analgesic pathway [31]. Propacetamol, however, blocks this enzyme at its peroxidase catalytic and not cyclooxygenase catalytic site and is not considered a NSAID. Riad and Moussa assessed the effects of lornoxicam on hemodynamic changes after tracheal intubation and found that administration of 8 mg of lornoxicam 30 minutes before surgery significantly decreased HR and BP subsequent laryngoscopy and tracheal intubation compared to placebo [32]. Lornoxicam, which is a novel nonsteroidal anti-inflammatory drug (NSAID), is more known for its postoperative pain reduction [33].

Lidocaine is used as a routine drug to relieve pain and cardiovascular responses to tracheal intubation in a considerable number of operating rooms. Recent studies have demonstrated that lidocaine alone or in combination with esmolol is not as effective as opioids on attenuating blood pressure and heart rate after intubation [14, 18]. Min et al., in a well-designed trial on 66 patients who underwent elective surgery, compared preventive effects of remifentanil 1 mg/kg and lidocaine 1.5 mg/kg + esmolol 1 mg/kg on hypertension and tachycardia after endotracheal intubation [14]. They revealed that remifentanil 1 mg/kg is more effective than the combination of lidocaine 1.5 mg/kg and esmolol 1 mg/kg for attenuating hemodynamic responses to rapid sequence intubation. Incidence rates of hypertension (3% versus 20%), hypotension (0.0% versus 20%), and tachycardia (3% versus 20%) were significantly lower in group P compared to group L. Feng et al. reported postintubation tachycardia (HR > 100 beat/min) and hypertension (SBP > 180 mmHg) in seventy-five percent of patients who received lidocaine [34]. In our trial, the definitions of tachycardia (HR > 120) and hypertension (MAP > 120 mmHg) were different, which might explain the observed differences. Further studies are required to assess various doses of propacetamol in order to compare it with other drugs like fentanyl, remifentanil, and esmolol or in combination with other drugs. The selected patients in our trial were between 20 and 60 years old and did not have any considerable predisposing factor, which may have affected their cardiovascular system. If potential benefits of propacetamol are confirmed, it will be possible to examine it in more specific cases, including patients with hypertension, cardiovascular diseases, the elderly, pregnant patients, and those with contraindication to other drugs.

## 5. Conclusion

Propacetamol 2 gr one hour prior intubation attenuates heart rate responses after laryngoscopy, but is not effective to prevent acute alterations in blood pressure after intubation.

## Figures and Tables

**Table 1:** Patients' baseline data in each group.

Variables	Propacetamol (*n* = 32)	Lidocaine (*n* = 30)	*P* value
Age (year)	43 ± 12	39 ± 11	0.2
ASA (I/II)	1/31	2/28	0.6
Sex (M/F)	15/17	13/17	0.8
Weight (kg)	74 ± 10	77 ± 10	0.3
Height (cm)	164 ± 15	166 ± 12	0.6
Endoscopic view (I/II)	20/12	19/11	0.9

**Table 2:** Hemodynamic variables including systolic, diastolic, and mean arterial blood pressures (SBP, DBP, MAP) and heart rate (HR) before and after drug in each group.

Variables	Propacetamol (*N* = 32)			Lidocaine (*N* = 30)		
	Before	After	*P* value	Before	After	*P* value
Systolic blood pressure (mmHg)	125 ± 11	112 ± 22	<0.001	126 ± 21	97 ± 21	<0.001
Diastolic blood pressure (mmHg)	82 ± 10	72 ± 9	<0.001	83 ± 14	65 ± 14	<0.001
Mean arterial blood pressure (mmHg)	96 ± 9	86 ± 11	<0.001	97 ± 15	75 ± 17	<0.001
Hear rate (beat/min)	81 ± 8	72 ± 5	<0.001	85 ± 17	89 ± 21	0.2
